# Validation of the Headache Impact Test (HIT-6) in patients with chronic migraine

**DOI:** 10.1186/s12955-014-0117-0

**Published:** 2014-08-01

**Authors:** Regina Rendas-Baum, Min Yang, Sepideh F Varon, Lisa M Bloudek, Ronald E DeGryse, Mark Kosinski

**Affiliations:** QualityMetric Incorporated, 24 Albion Road, Bldg 400, Lincoln, RI 02865-4207 USA; Allergan, Inc., 2525 Dupont Drive, Irvine, CA 92612 USA; Xcenda, LLC., 4114 Woodlands Pkwy, Palm Harbor, FL 34685 USA

**Keywords:** Headache Impact Test (HIT-6), Psychometrics, Validity, Chronic migraine, Health-related quality of life, PREEMPT

## Abstract

**Background:**

The Headache Impact Test (HIT)-6 was developed and has been validated in patients with various types of headache. The objective of this study was to report the psychometric properties of the HIT-6 among patients with chronic migraine.

**Methods:**

Data came from two international, multicenter, randomized, double-blind, placebo-controlled clinical trials of chronic migraine patients (N = 1,384) undergoing prophylaxis therapy. Confirmatory factor analysis and differential item functioning (DIF) analysis were used to test the latent structure and cross-cultural comparability of the HIT-6. Reliability, construct validity, and responsiveness were assessed. Two sets of criterion groups were used: (1) 28-day headache frequency: <10, 10–14, and ≥15 days; (2) sample quartiles of the total cumulative hours of headache: <140, 140 to <280, 280 to <420, and ≥420 hours. Two sets of responsiveness categories were defined as reduction of <30%, 30% to <50%, or ≥50% in (1) number of headache days and (2) cumulative hours of headache.

**Results:**

Measurement invariance tests supported the stability of the HIT-6 latent structure across studies. DIF analysis supported cross-cultural comparability. Good reliability was observed across studies (Cronbach’s α: 0.75–0.92; intraclass correlation coefficient: 0.76–0.80). HIT-6 scores correlated strongly (−0.86 to −0.59) with scores of the Migraine-Specific Quality-of-Life Questionnaire. Analysis of variance indicated that HIT-6 scores discriminated across both types of criterion groups (*P*<0.001), across studies and time points. HIT-6 change scores were significantly higher in magnitude in groups experiencing greater improvement (*P*<0.001).

**Conclusion:**

All measurement properties were consistently verified across the two studies, supporting the validity of the HIT-6 among chronic migraine patients.

**Trial registration:**

NCT00156910 and NCT00168428 on www.ClinicalTrials.gov.

## Background

Migraine is recognized as a major cause of disability, adversely affecting the daily functioning of an estimated 12% of the US population [1,2]. For some patients, the frequency of migraine attacks increases over time and progresses to high-frequency episodic migraine (10–14 days per month) or chronic migraine (≥15 days per month) [3]. Patients with episodic migraine (<15 days per month) are estimated to transition into chronic migraine at a rate of about 2.5% per year [4]. Increases in the frequency of headache leading to chronic migraine are known to be accompanied by anatomical and functional changes [5]. Understanding the impact of these changes helps define the characteristics of chronic migraine patients and how they differ from those of patients with lower frequency migraine. Frequency of headaches is the key distinguishing feature used to classify migraine subtypes (e.g., low-frequency episodic migraine, high-frequency episodic migraine, and chronic migraine) [6]; however, researchers have become increasingly aware that the differential impact of migraine cannot be fully assessed through the exclusive use of such measures. This has led to the increasing use of disease-specific patient-reported outcome (PRO) measures in migraine research and clinical practice [7]. The use of standardized PRO instruments has contributed greatly to a more accurate assessment of the impact of the disorder on patients’ health-related quality of life (HRQOL), and these measures are currently recommended by experts in the field [8,9]. Nevertheless, the integrity of PRO-based evaluations rests on the availability of psychometric data endorsing its validity among the clinical population of interest. Several disease-specific measures of quality of life were developed and validated in samples of patients with headache. PRO instruments measuring headache impact are often used across different migraine subtypes, the assumption being that the instrument’s validity is retained across these clinical subgroups. However, evidence of an instrument’s properties among specific subtypes of migraine is often not available.

The 6-item Headache Impact Test (HIT-6™) is a brief tool for assessing the impact of headache in both clinical research and practice. The development and validation study indicated that the HIT-6 possessed good psychometric properties among headache sufferers [10]. Another study conducted among patients seeking headache-specialty care confirmed high indices of reliability, as well as construct and face validity [11]. Several important properties of the HIT-6 have been documented, including its between-group and within-group minimally important difference (MID) [12,13] and its ability to detect change in clinical measures of migraine patients [14]. Although strong evidence has been found supporting the validity of the HIT-6 in headache sufferers in general, a full evaluation of the HIT-6 psychometric properties specifically in a subgroup of headache patients with chronic migraine, a distinct headache disorder characterized by the International Classification of Headache Disorders Revised Criteria (ICHD-IIR) as ≥15 headache days per month for at least 3 months, with ≥8 days per month fulfilling criteria for migraine without aura, has not been conducted. The ability to detect significant change in clinical parameters [14,15] has been evaluated in samples of patients with chronic daily headache or chronic migraine, but a full psychometric evaluation, including longitudinal measurement invariance and differential item functioning, was not performed. Furthermore, none of the previous validation studies [10,11] were conducted using clinical trial samples. This is an important aspect of validity because PRO instruments are key elements in efficacy studies of migraine treatment. The current study used data from two independent clinical trials of chronic migraine patients to comprehensively evaluate the psychometric properties of the HIT-6 in chronic migraine patients.

## Methods

### Sample

Data used in the analyses came from a total sample of 1,384 patients with chronic migraine who participated in 2 studies that evaluated onabotulinumtoxinA (BOTOX®, Allergan, Inc.) as headache prophylaxis – the Phase III REsearch Evaluating Migraine Prophylaxis Therapy with Botulinum Toxin Type A (PREEMPT) trials [16,17]. Both PREEMPT trials were multicenter, double-blind, randomized, and placebo-controlled. The total study period consisted of 60 weeks, which included a 4-week baseline phase capturing inclusion/exclusion information, followed by a 24-week double-blind treatment phase, and a final 32-week, open-label extension phase. The studies were conducted between January of 2006 and August of 2008 in the United States, Canada, the United Kingdom, Croatia, Germany and Switzerland.

To be considered eligible for the trial, participants had to be between the ages of 18 and 65 years and fulfill each of the following headache-related criteria: 1) history of migraine headache disorder meeting any of the diagnostic criteria listed in ICHD-II [18] section 1, for migraine, with the exception of “complicated migraine”; 2) ≥4 distinct headache episodes each with a duration of at least 4 hours during the 4-week baseline phase; 3) ≥15 headache days during the 4-week baseline phase, with each headache day consisting of ≥4 hours of continuous headache; and 4) ≥50% of baseline headache days were migraine or probable migraine days. Headache-related exclusion criteria included any of the following criteria: 1) diagnosis of complicated migraine, basilar migraine, ophthalmoplegic migraine, or migrainous infarction; 2) use of any headache prophylactic medication within 28 days prior to screening; 3) diagnosis of chronic tension-type headache, hypnic headache, hemicrania continua, or new daily persistent headache; 4) headache attributed to another disorder (e.g., cervical dystonia, craniotomy, head/neck trauma); and 5) unremitting headache lasting continuously throughout the 4-week baseline period. In addition, participants with a Beck Depression Inventory score >24 at week 4 baseline period were also excluded.

Data from the 24-week, double-blinded period of the two trials were used for the current study. All analyses were conducted by pooling treatment groups.

### Measures

#### HIT-6

The items of the HIT-6 cover several HRQOL domains: pain, social functioning, role functioning, vitality, cognitive functioning, and psychological distress. Each item is answered on a 5-point Likert scale (6 = never, 8 = rarely, 10 = sometimes, 11 = very often, 13 = always). The scoring of the HIT-6 was derived to approximate the total score obtained from a larger battery of items, using results from item response theory (IRT) [10]. The final score is obtained from simple summation of the six items. The HIT-6 total score ranges between 36 and 78, with larger scores reflecting greater impact. Four groups have been derived to aid in the interpretation of HIT-6 scores: scores ≤49 represent little or no impact; scores between 50 and 55 represent some impact; scores between 56 and 59 represent substantial impact; and scores ≥60 indicate severe impact [19]. The HIT-6 was administered to study participants at each office visit of the double-blind phase: at baseline and every 4 weeks up to Week 24.

#### Migraine-specific questionnaire

The Migraine-Specific Quality-of-Life Questionnaire (MSQ) is a relatively longer questionnaire compared to the HIT-6 and is used to assess the impact of migraine on the HRQOL of migraine patients [20,21]. In its most current version (version 2.1), the MSQ is composed of 14 items, each measured on a 6-point scale (1 = none of the time, 6 = all of the time), with higher scores reflecting greater impact. The MSQ measures the impact of migraine on the patient’s HRQOL in the past 4 weeks across three dimensions: role function-restrictive (7 items), role function-preventive (4 items), and emotional function (3 items). Raw dimension scores are computed as a sum of item responses and rescaled to a 0–100 scale with severity direction reversed (higher scores represent better migraine-specific quality of life). Study participants were asked to answer the MSQ at baseline, Week 12, and Week 24.

#### Migraine diary

Using a self-administered diary, participants were asked to report information on the timing of headaches, headache-specific characteristics, symptoms, and use of any acute headache pain medication. A headache day was defined as a day with 4 or more continuous hours of headache. A migraine day was defined as a day with 4 or more continuous hours of migraine headache (ICHD-II criteria for migraine without aura or migraine with aura). A probable migraine day was defined as a day with 4 or more continuous hours of probable migraine headache (ICHD-II for probable migraine).

### Statistical analyses

The psychometric evaluation of the HIT-6 was conducted in a sequential process. First, analyses were conducted to test the comparability of the two study samples and evaluate the adequacy of analytical approaches that may be sensitive to distributional characteristics. Specifically, the chi-square test was used to detect differences in categorical variables while the parallel-group *t*-test was used to detect differences in continuous variables. Next, several analyses were conducted to ensure the stability of the HIT-6 measurement model. Confirmatory factor analysis (CFA) was employed in order to ensure consistency with the HIT-6 measurement model. Multi-group CFA was used to conduct tests of measurement invariance across the two clinical trials and longitudinal measurement invariance. Testing for differential item functioning (DIF) was conducted to establish the cross-cultural comparability of HIT-6 scores from participants in the United States, Canada, and four European countries (Croatia, Germany, Switzerland, and United Kingdom). Upon the verification of the stability of the HIT-6 measurement model, item-level psychometric indicators were examined, followed by an evaluation of the instrument’s reliability, construct validity, and ability to detect change.

#### Structural validity

The latent structure of the HIT-6 was examined under CFA using baseline. Consistent with a single dominant trait measurement model [10,19], our hypothesis was that the factor loadings of a one-factor model would be at least moderate in magnitude (>0.50) and similar across items. Multi-group categorical CFA was then employed to examine whether the measurement model of the HIT-6 was invariant across the two studies. Invariance testing was conducted by imposing a series of hierarchical equality constraints across these two samples. Using the guidelines provided by Millsap and Yun-Tein [22], we began by fitting a model in which all parameters (except the loading of the first item, which was set equal to 1 for model identification purposes) were freely estimated across studies (configural invariance). Invariance testing proceeded by comparing the chi-square value of this model to a model where item thresholds were constrained to equality across studies (scalar invariance). A third model was then fit in which both item thresholds and loadings (metric invariance) were constrained to equality across groups, and its chi-square value was compared to that of the scalar invariance model. The last comparison was made between the metric invariance model and a model that further restricted residual variances to be equal across groups. Invariance was evaluated by comparing the chi-square values from nested models where parameters were fixed across studies [23]. At each step, invariance was verified if the model with the greater number of constraints was not significantly different from the initial model. If the full sequence of invariance tests was verified, then total measurement invariance across studies was deemed to be present. CFA was conducted using the robust weighted least squares estimator as implemented in MPlus (version 5.1) [24]. The CFA model fit was assessed using several indicators: comparative fit index (CFI), Tucker-Lewis Index (TLI), root mean square error of approximation (RMSEA), and weighted root mean residual (WRMR). Hu and Bentler’s [18] guidelines were used to interpret the values of CFI and TLI (≥.95), RMSEA (<.06), and WRMR (<.90), indicating good fit.

The cross-cultural comparability of HIT-6 scores was investigated by examining measurement invariance across country- or region-specific groups of patients. The sample sizes of the four European countries were insufficient to carry out country-specific analyses of measurement invariance. Hence, our analysis was based on the following groups: United States, Canada, and pooled European countries. DIF tests were carried out under the method of Crane, Gibbons, and Jolley [25]. Under this method, ordinal logistic regression models are fit to each item, using group membership and trait level as the explanatory variables. To take into account current expert recommendations [26], two alternative trait estimates were used: (1) factor scores estimated from the ordinal CFA (configural model) and (2) HIT-6 sum scores. For each type of trait estimate, the following sequential process was used to test for the presence of DIF: first, we tested the presence of item bias throughout the trait continuum (or uniform DIF) by examining the change in the trait level coefficient brought about by removing the group membership term from the model. Changes to the trait-level coefficient higher than 10% were indicative of item bias. Results of simulation studies have shown this latter criterion to be superior to the 0.05 threshold for statistical significance of the trait-level coefficient [27]. Second, we tested whether the relationship between migraine impact and item scores was dependent on country group (non-uniform DIF). This test was conducted by evaluating whether the interaction term between group membership and trait level was significant at a confidence level equal to 0.0083 (=0.05/6). In accordance with the approach of Crane and colleagues [25], a Bonferroni correction for multiple comparisons was applied, due to testing for each of the 6 items of HIT-6. The assumption of proportional odds was thought to be violated if the *P*-value for the score test was below 0.05 and the plots of the empirical logits indicated nonparallel lines [28].

#### Reliability

Indices of reliability reflect the consistency and reproducibility of scores produced by a particular measurement procedure. Two distinct methods were used to estimate reliability: (1) test-retest reliability was evaluated by correlating scores from one administration with scores from another administration, for participants who self-reported stable migraine symptoms across administrations; and (2) internal consistency reliability was evaluated by examining the equivalence of responses in a single administration. To evaluate test-retest reliability, a “stable” subsample was first identified at Study Weeks 8 and 12. Participants were considered stable across these two time points if, at Week 12, they answered “my migraine symptoms are the same” to the question “What effect has your current medication(s) had on your migraine symptoms in the past 4 weeks?” (item 2 of the Migraine Treatment Satisfaction Questionnaire). The intra-class correlation coefficient (ICC) was then evaluated among the stable subsample in each study and interpreted using established criteria [29]. Internal consistency reliability of the HIT-6 at baseline and Week 24 was measured with three indices: Cronbach’s alpha, the average inter-item correlation [30], and the item-total correlation after correcting for overlap, i.e., after removing the item from the total score. Cronbach’s alpha was evaluated against currently recommended criteria [31]. Item-total correlations and average inter-item correlations of 0.4 or higher were deemed indicative of good reliability [32].

#### Construct validity

The convergent validity of the HIT-6 scores was assessed in relation to MSQ scores. Correlation coefficients, evaluated at baseline and at Week 24, were interpreted as indicative of convergent validity if they were < −0.40. The negative sign reflects the fact that while higher scores in the HIT-6 are indicative of greater headache impact, higher scores on the MSQ are indicative of better HRQOL.

Construct validity was also examined using the framework of known-groups validity [33]. This approach consists of comparing mean scale scores across groups known to differ on a clinical criterion measure. In the present study, groups were based on the following clinical indicators of chronic migraine: 1) number of headache days within a 28-day period and 2) cumulative hours of headache within a 28-day period. Drawing on classification criteria previously used in migraine research [34], participants were classified into one of three headache frequency categories: <10 headache days, 10–14 headache days, or ≥15 headache days. In addition, four groups were formed based on quartiles of the sample’s (combined study 1 and study 2) distribution of cumulative hours of headache: 1) <140 hours, 2) 140 to <280 hours, 3) 280 to <420 hours, and 4) ≥420 hours. These cutoffs corresponded to an average of approximately <5 hours, 5–10 hours, >10–15 hours, and ≥15 hours of headache per day, respectively. Because eligibility required study participants to have a minimum of 15 headache days in a 28-day period, baseline data were not used in these analyses. Therefore, known-groups validity analyses were performed using data from Week 24.

#### Responsiveness

The responsiveness of the HIT-6 was evaluated against changes (baseline to Week 24) in number of headache days and cumulative hours of headache in the pooled onabotulinumtoxinA arms of both studies. Participants were categorized according to the direction and magnitude of change in these measures. In agreement with recommendations of the Task Force of the International Headache Society Clinical Trials Subcommittee [8], a subject was categorized as “much improved” if the 28-day frequency of headache days decreased by ≥50%; as “moderately improved” if this decrease was ≥30% but <50%; and as “not improved” if this decrease was <30% or if worsening was reported. A similar categorization scheme was applied to our second criterion measure, cumulative hours of headache. Due to the small number of study participants reporting worsening of either frequency of headache days or cumulative hours of headache, worsening was combined with improvement that was not deemed significant (<30%). HIT-6 change scores were found to be approximately normally distributed supporting the use of analysis of variance (ANOVA) models to evaluate whether group differences in mean HIT-6 change score were statistically significant. The standardized response mean (SRM), which is a measure of effect size calculated as the ratio of the mean HIT-6 change score to its standard deviation, was evaluated to help interpret the magnitude of change across the three improvement groups defined above.

## Results

### Sample characteristics

Table 1 presents the main demographic and clinical characteristics separately for each study sample. Of 1,384 patients enrolled in the two studies, 1,376 had HIT-6 scores at baseline. Overall, study participants were primarily female and Caucasian, and had an average age of approximately 41 years. Based on patients’ baseline assessment, the average number of migraine days in a 28-day period was approximately 16 (19 when probable migraine days were included), the average number of headache days was approximately 20, and the average cumulative hours of headache in the 28 day period was approximately 288.

**Table 1:** **Characteristics of study participants at baseline (N = 1**,**384)**

**Characteristics**	**Study 1**		**Study 2**	
	**(n = 679)**		**(n = 705)**	
Age (years), mean (SD)	41.7	(10.5)	41.0	(10.6)
Gender, n (% female)	594	(87.5)	602	(85.4)
Race				
Caucasian	614	(90.4)	633	(89.8)
Black	30	(4.4)	44	(6.2)
Hispanic	29	(4.3)	17	(2.4)
Asian	3	(0.4)	4	(0.6)
Other	3	(0.4)	7	(1.0)
Migraine Characteristics				
Years since frequent migraine onset, mean (SD)	20.4	(13.0)	18.0	(12.1)
Number of headache days during the 28 day baseline period, mean (SD)	19.9	(3.7)	19.8	(3.6)
Number of migraine days in a 28-day period, mean (SD)	16.5	(5.8)	16.3	(5.8)
Number of migraine/probable migraine days in a 28-day period, mean (SD)	19.1	(4.0)	18.9	(4.0)
Cumulative hours of headache in a 28-day period, mean (SD)	285.3	(114.3)	291.6	(119.6)
HIT-6^a^, mean (SD)	65.6	(4.0)	65.3	(4.4)
MSQ^b^				
Role function - preventive	55.4	(21.0)	56.7	(21.9)
Role function - restrictive	37.8	(16.8)	39.4	(17.0)
Emotional function	40.3	(24.1)	44.1	(24.8)

^a^HIT-6 varies between 36 and 78, with higher scores indicating higher headache impact. Of the 1,384 patients enrolled, 1,376 participants (n = 672 for Study 1 and n = 704 for Study 2) had HIT-6 scores at baseline.

^b^The MSQ total and scale score varies between 0 and 100, with higher scores indicating less impact.

HIT-6 = Headache Impact Test-6; MSQ = Migraine-Specific Questionnaire.

Baseline scores on the HIT-6 and MSQ were nearly identical across the two studies. At baseline, the average HIT-6 score was approximately 65 (65.6 and 65.3 for studies 1 and 2, respectively), reflecting a severe level of headache impact [19]. Scores on the MSQ were also reflective of severe impact. The order of increasing severity was constant across the two studies, with migraine-attributable interruptions in daily activities (role-preventive dimension) reflecting the lowest impact and limitations due to migraine (role-restrictive dimension) being the most severely affected of the three MSQ dimensions.

The demographic and migraine characteristics of patients were nearly identical across studies, as were the distributions of scores for each HIT-6 item. At baseline, about 30% of patients reported that they frequently wished they could lie down. Approximately the same proportion also stated that they frequently felt irritated because of their headaches. These two items stood out as those occurring with greatest frequency. For each of the remaining four items (pain, daily activities interference, too tired to do work/daily activities, and ability to concentrate), between 5% and 8% of patients said it occurred frequently. Nevertheless, about 62% of the overall sample said headaches very often limited their ability to concentrate.

### Structural validity

The standardized loadings obtained under the configural model (Table 2) indicated that, across the two studies, all HIT-6 items were similarly strongly correlated with headache impact. Restricting model parameters to equality across the two studies did not result in a significant deterioration of model fit, as measured by the chi-square test for nested models (Table 3). Indeed, the values shown in Table 3 for TLI, CFI, and RMSEA suggest that a slightly better fit is obtained under total measurement invariance across studies. These results provide evidence that the measurement model of the HIT-6 remained stable across two independent samples of chronic migraine patients. Tests of measurement invariance across country groups (Table 4) showed no evidence of uniform or non-uniform DIF, as indicated by the small changes in the value of the trait level coefficient after removal and likelihood-ratio tests comparing the models with and without the trait-level group membership interaction, respectively. In addition, results (not shown) using the HIT-6 sum score as the trait level variable (instead of CFA factor scores) resulted in identical conclusions. Inspection of the score test in conjunction with plots of the empirical logits suggested that the proportional odds assumption was not met by the item “In the past 4 weeks, how often have you felt fed up or irritated because of your headaches?” Nevertheless, the degree of non-parallelism shown in the plot was small (results not shown).

**Table 2:** **Standardized factor loadings from confirmatory factor analysis** ^*^

**HIT-6 item**		**Study 1**		**Study 2**	
		**(n = 672)**		**(n = 702** ^**a**^ **)**	
		**Factor loading**	**Standard error**	**Factor loading**	**Standard error**
A^b^	*Pain Severe When Headache*	0.652	(0.032)	0.740	(0.025)
B	*Limit Ability to Do Daily Acts*	0.770	(0.023)	0.797	(0.020)
C	*Wish Could Lie Down*	0.536	(0.032)	0.658	(0.028)
D	*Too Tired to Do Work or Daily Acts*	0.785	(0.022)	0.796	(0.021)
E	*Feel Fed Up/Irritated Because of Headache*	0.533	(0.033)	0.554	(0.031)
F	*Limit Ability to Concentrate*	0.751	(0.027)	0.744	(0.024)

^a^Only includes participants with complete item level HIT-6 data.

^b^Factor loading fixed to one for identification purposes.

RMSEA = 0.086; WRMR = 1.263; CFI/TLI = 0.976/0.978.

CFI = comparative fit index; RMSEA = root mean square error of approximation; TLI = Tucker-Lewis Index; WRMR = weighted root mean square residual.

^*^Configural Invariance Model at Baseline.

**Table 3:** **Tests of measurement invariance using multi-group confirmatory factor analysis**

	**Model fit**			**Model comparison**		
**Parameter constraints**	**TLI**	**CFI**	**RMSEA**	**∆CFI**	**∆RMSEA**	**Chi-square for nested model comparison**
**Measurement Invariance Across Studies**						
None - Configural Model	0.979	0.978	0.086	—	—	—
Thresholds	0.990	0.980	0.059	0.002	−0.027	22.754 (DF = 15); (*P* = 0.0895)
Thresholds and loadings	0.992	0.982	0.052	0.002	−0.007	3.579 (DF = 4); (*P* = 0.4659)
Thresholds, loadings, and residual variances	0.993	0.982	0.050	0.000	−0.002	9.655 (DF = 6); (*P* = 0.1399)

Values under the column heading “Model Comparison” refer to the comparison of the model in the corresponding row and the model in the preceding row.

CFI = comparative fit index; ΔCFI = change in CFI; DF = degrees of freedom; RMSEA = root mean square error of approximation; ΔRMSEA = change in RMSEA; TLI = Tucker-Lewis Index.

**Table 4:** **Tests of measurement invariance across country groups**

**HIT-6 item**		**N**	**Percent change in the value of the coefficient** ^**a**^	Significance testing for interaction between country and trait level*		
				**Wald chi-square**	**DF**	***P*** **-value**
A	*Pain Severe When Headache*	1,375	0.2	5.187	2	0.0747
B	*Limit Ability to Do Daily Acts*	1,376	2.8	6.402	2	0.0407
C	*Wish Could Lie Down*	1,376	2.2	1.847	2	0.3971
D	*Too Tired to Do Work or Daily Acts*	1,376	0.1	1.241	2	0.5377
E	*Feel Fed Up/Irritated Because of Headache*	1,375	7.8	2.243	2	0.3258
F	*Limit Ability to Concentrate*	1,376	1.4	2.362	2	0.3070

*P-values <0.0083 (=0.05/6) indicate non-uniform DIF.

^a^Percent change in the trait level coefficient after removing the country group indicators from the model; values ≥10% indicate uniform DIF.

DF = degrees of freedom; DIF = differential item functioning.

### Reliability

Study-specific estimates of the ICC, used to measure test-retest reliability, were generally indicative of good reliability (Table 5). The ICC was 0.80 (95% confidence interval [CI] = [0.75, 0.83]) for study 1 and 0.76 (95% CI = [0.72, 0.80]) for study 2 (Table 5). Cronbach’s alpha was consistently above the recommended threshold for acceptable reliability (0.70) and close to values indicating good to excellent reliability (>0.80). The relative contribution of each item to the scale’s internal consistency was assessed by evaluating alpha-removed statistics (results not shown). The magnitude of change in Cronbach’s alpha was nearly uniform across items, and in no instances did removal of an item from the scale result in an increase in the value of Cronbach’s alpha. Item-total correlations of 0.40 or higher were observed for all items across time and studies, supporting the validity of each item to the total scale. At baseline, the average inter-item correlation was 0.45 for study 1 and 0.53 for study 2. Both values are higher than the recommended threshold of 0.40 [32]. Overall, using recommended interpretation guidelines, measures of reliability were homogeneously supportive of the hypothesis of consistent and reproducible HIT-6 scores among the two samples of chronic migraine patients.

**Table 5:** **Measures of reliability for the HIT-6**

**Study**	**N** ^**a**^	**ICC** ^**b**^	**95%** **confidence interval**	**Cronbach’s alpha** ^**c**^		**Average inter-item polychoric correlation** ^**c**^	
				**Baseline**	**Week 24**	**Baseline**	**Week 24**
1	362	0.80	[0.75, 0.83]	0.75	0.92	0.45	0.73
2	349	0.76	[0.72, 0.80]	0.79	0.91	0.53	0.72

^a^Participants who, at Week 12, answered “my migraine symptoms are the same” to the question “What effect has your current medication(s) had on your migraine symptoms in the past 4 weeks?”.

^b^ICC = intra-class correlation coefficient; estimated for measurements made at Weeks 8 and 12.

^c^Study 1: N _baseline_ = 672; N _week 24_ = 580; study 2: N _baseline_ = 702; N _week 24_ = 637.

HIT-6 = Headache Impact Test-6.

### Construct validity

Correlations between HIT-6 total scores and scale scores of the MSQ (absolute values) were above the recommended threshold of 0.40 for convergent validity [32] across studies and time points, ranging between −0.86 and −0.59 (Table 6), suggesting good convergent validity.

**Table 6:** **Convergent validity: correlations between HIT-6 and MSQ scores**

**Time point**	**Item/Scale**	**Study 1**		**Study 2**	
		**Correlation** ^**a**^	**N**	**Correlation** ^**a**^	**N**
Baseline	Total	−0.77	(671)	−0.78	(703)
	Emotional function	−0.62	(671)	−0.59	(703)
	Role function - preventive	−0.67	(672)	−0.66	(704)
	Role function - restrictive	−0.75	(672)	−0.78	(704)
Week 24	Total	−0.85	(576)	−0.83	(637)
	Emotional function	−0.78	(576)	−0.74	(637)
	Role function - preventive	−0.74	(579)	−0.74	(637)
	Role function - restrictive	−0.86	(580)	−0.84	(637)

^a^Pearson product moment correlations.

HIT-6 = Headache Impact Test-6; MSQ = Migraine-Specific Questionnaire.

Known-groups validity analyses were supportive of the validity of the HIT-6 scores with respect to clinical criterion measures (Table 7). For study 1, at Week 24, mean HIT-6 scores were significantly different across levels of headache frequency (*P*-value <0.001), with values equal to 58.4, 62.9 and 65.0, for individuals experiencing <10, 10–14, and ≥15 days of headache, respectively. Very similar results were observed in study 2. Further, mean HIT-6 scores were higher in groups indicating greater impact of migraine, as represented by quartiles of cumulative hours of headache. These results were consistent across the two studies.

**Table 7:** **Known-groups validity: HIT-6 scores at week 24 in relation to clinical criterion measures**

***Number of headache days***														
	**<10 Days**			**10–14 Days**			**≥15 Days**							
**Study**	**Mean**	**SD**	**N**	**Mean**	**SD**	**N**	**Mean**	**SD**	**N**				**F** ^**a**^	**F** ^**b**^
1	58.4	(8.18)	257	62.9	(5.48)	154	65.0	(5.70)	268				125.02*	125.28*
2	58.2	(8.21)	288	63.3	(6.33)	171	64.6	(5.17)	246				120.71*	114.87*
***Cumulative Hours of Headache***														
	**Quartile 1: <140 hours**			**Quartile 2: 140 to <280 hours**			**Quartile 3: 280 to <420 hours**			**Quartile 4: ≥420 hours**				
**Study**	**Mean**	**SD**	**N**	**Mean**	**SD**	**N**	**Mean**	**SD**	**N**	**Mean**	**SD**	**N**	**F** ^**a**^	**F** ^**b**^
1	59.1	(7.74)	313	63.6	(5.78)	186	65.5	(5.27)	106	65.9	(6.27)	74	105.97*	110.12*
2	59.2	(8.08)	357	63.5	(5.80)	199	64.8	(5.15)	90	66.0	(5.49)	59	89.50*	88.38*

*P-value <0.001.

^a^F-Statistics and *P*-values for between-category comparisons are from analysis of variance (ANOVA). The main effect in the ANOVA was ranked category of decrease in the clinical criterion measure (i.e., headache days or cumulative hours), where the type III sum of squares was used.

^b^F-Statistics and *P*-values for between-category comparisons are from ANOVA. The main effects in the ANOVA included ranked category of decrease in the clinical criterion measure (i.e., headache days or cumulative hours) and medication-overuse strata, where the type III sum of squares was used.

HIT-6 = Headache Impact Test-6; SD = standard deviation.

### Responsiveness

Across both studies, the mean HIT-6 change score was significantly greater for groups of patients representing greater degree of improvement in frequency of headache days and cumulative hours of headache (Table 8). On average, patients experiencing at least a 50% improvement in the number of headache days reported a nearly 7-point decrease in HIT-6 score, far exceeding the established MID of a 2.3-point decrease [12]. Patients who experienced moderate improvement reported an average decrease of 3.3 and 2.9 points, for studies 1 and 2, respectively, while the mean decrease in HIT-6 scores for patients experiencing less than a 30% reduction in the number of headache days was −0.7, for both studies. Differences in mean HIT-6 change scores were similar across groups of patients representing greater degree of improvement in cumulative hours of headache. Using the guidelines of Cohen [35], these results indicate large (0.8), medium (0.5), and low (0.2) effect sizes for highest (≥50%), moderate (≥30% to <50%), and lowest (<30%) categories of improvement. The gradient of change across groups of improvement for both criterion variables suggests that the HIT-6 appropriately captures changes in migraine frequency in chronic migraine patients.

**Table 8:** **Responsiveness: change in HIT-6 scores in relation to changes in clinical criterion measures**

**Decrease from baseline in headache days at week 24**						
	**Mean change in HIT-6**	**SD**	**n**	**SRM**	**F-value** ^**a**^	**F-value** ^**b**^
Study 1						
≥50%	−6.9	7.63	255	−0.90		
≥30% to <50%	−3.3	5.18	141	−0.64		
<30%	−0.7	4.23	283	−0.17	148.81*	148.33*
Study 2						
≥50%	−6.9	8.07	291	−0.86		
≥30% to <50%	−2.9	5.60	131	−0.52		
<30%	−0.7	4.01	283	−0.18	140.01*	139.62*
**Decrease from Baseline in Cumulative Hours of Headache Occurring on Headache Days at Week 24**						
	**Mean Change in HIT-6**	**SD**	**n**	**SRM**	**F-value** ^**a**^	**F-value** ^**b**^
Study 1						
≥50%	−6.9	7.50	269	−0.92		
≥30% to <50%	−2.6	5.24	115	−0.50		
<30%	−0.9	4.30	295	−0.21	142.06*	143.15*
Study 2						
≥50%	−6.5	8.05	319	−0.81		
≥30% to <50%	−2.2	4.64	109	−0.47		
<30%	−0.9	4.41	277	−0.20	112.72*	112.07*

*P-value <0.001.

^a^F-Statistics and *P*-values for between-category comparisons are from analysis of variance (ANOVA). The main effect in the ANOVA was ranked category of decrease in the clinical criterion measure (i.e., headache days or cumulative hours), where the type III sum of squares was used.

^b^F-Statistics and *P*-values for between-category comparisons are from analysis of variance (ANOVA). The main effects in the ANOVA included ranked category of decrease in the clinical criterion measure (i.e., headache days or cumulative hours) and medication-overuse strata, where the type III sum of squares was used.

HIT-6 = Headache Impact Test-6; SD = standard deviation; SRM = standardized response mean.

## Discussion

The findings of the current study support the stability of the HIT-6 measurement model, the tool’s construct validity, and its ability to detect change in clinical indicators of headache in two independent samples of chronic migraine patients undergoing treatment. Using recommended guidelines for interpretation of change in clinical indicators of headache [8], we found that the HIT-6 captured different levels of change exceptionally well, as indicated by measures of effect size. Indeed, the magnitude and direction of mean HIT-6 change scores were fully in line with the percentage improvement in headache frequency reported by patients, with strong, moderate, and low effects sizes for the highest (>50%), middle (30% to 50%), and lowest (<30%) categories of improvement, respectively. Improvement of at least 50% in headache day frequency was accompanied by an average increase in HIT-6 scores of approximately 7 points, which substantially exceeds previous estimates of patients’ perceptions of meaningful change [12]. Our findings concerning the reliability and construct validity of the HIT-6 also demonstrate that the instrument has excellent properties in this respect. Previous studies [10,11] reported internal consistency estimates for the HIT-6 that ranged between 0.89 and 0.90, a result that was consistent with the one found in our study. We found similar agreement between our estimates of test-retest scale reliability (the ICC was equal to 0.80 for study 1 and 0.76 for study 2) and those reported in the HIT-6 validation study [10]. The convergent validity of the HIT-6 was also supported by the finding of strong correlations (−0.9 to −0.6) with the MSQ. Construct validity was also evaluated using data from Week 24 in a known-groups framework. The results of these analyses showed that HIT-6 scores can be used to discriminate between patients with low-frequency episodic migraine (<10 days), high-frequency episodic migraine (10–14 days), and chronic migraine (≥15 days), a result that reinforces the findings of a previous study [15].

Some limitations should be taken into account in the interpretation of the study’s findings. First, the patient sample was taken from two clinical trials, therefore generalizability to the general population of chronic migraine patients may be limited. Second, the sample is representative of those migraine patients receiving onabotulinumtoxinA as prophylactic treatment and may not be generalizable to other migraine treatments. Nevertheless, CFA results were similar to those of analyses carried out in a sample of patients seen in a headache-specialty practice [11], as were other psychometric properties previously estimated among a general population of headache sufferers [10] and more recently in both episodic and chronic migraine patients [15]. Finally, although we found no evidence of item bias across the country groups examined, our sample did not allow for single country comparisons. Further, the number of observations in some groups may have limited the ability to detect mild or moderate DIF in the HIT-6 items. Although it is known that the estimation of ordinal logistic regression parameters is affected by the size of the sample, a recent study [36] examining the effect of sample size on the power to detect DIF indicated that when the group size is equal to 100 and the number of items is 6, ordinal logistic regression can detect strong DIF with high power (>90%), although power to detect moderate DIF is considerably lower (<60%). In the current study, two of the language groups had sample sizes of approximately 100, thus suggesting that moderate or mild DIF might have not been detected among these groups. In addition, the statistical significance tests indicated that the proportional odds assumption was not met by one of the six items of the HIT-6. As extensively documented [37,38], the score test is known to result in small *P*-values, even when the departure from the proportional odds assumption is not significant from a practical perspective. Although this finding affected a single item of the HIT-6 and the degree to which the assumption was violated appeared to be small upon visual inspection, studies with larger sample sizes across different countries are warranted to verify the absence of cross-cultural DIF for the HIT-6. Importantly, our findings agree with a previous study in which the psychometric properties of the HIT-6 were found to be similar across 11 languages [39].

## Conclusions

The evidence presented in the current study and its consistency with results from prior studies suggest that the HIT-6 has excellent properties to measure the impact of headache across a wide spectrum of headache frequency, including chronic migraine, and in different clinical and research settings.

## Abbreviations

ANOVA: Analysis of variance
CFA: Confirmatory factor analysis
CFI: Comparative fit index
DIF: Differential item functioning
HIT-6: 6-item Headache Impact Test
HRQOL: Health-related quality of life
ICC: Intra-class correlation coefficient
ICHD: International Classification of Headache Disorders
IRT: Item response theory
MID: Minimally important difference
MSQ: Migraine-Specific Quality-of-Life Questionnaire
PREEMPT: Phase III REsearch Evaluating Migraine Prophylaxis Therapy with Botulinum Toxin Type A
PRO: Patient-reported outcome
RMSEA: Root mean square error of approximation
SRM: Standardized response mean
TLI: Tucker-Lewis Index
WRMR: Weighted root mean residual
